# PI3Kδ Inhibition as a Potential Therapeutic Target in COVID-19

**DOI:** 10.3389/fimmu.2020.02094

**Published:** 2020-08-21

**Authors:** Giuseppe Palma, Teresa Pasqua, Giovannino Silvestri, Carmine Rocca, Paola Gualtieri, Antonio Barbieri, Anna De Bartolo, Antonino De Lorenzo, Tommaso Angelone, Ennio Avolio, Gerardo Botti

**Affiliations:** ^1^SSD Sperimentazione Animale, Istituto Nazionale Tumori Fondazione G. Pascale – IRCSS, Naples, Italy; ^2^Laboratory of Cellular and Molecular Cardiovascular Patho-Physiology, Department of Biology, Ecology and Earth Science, University of Calabria, Rende, Italy; ^3^Institute of Human Virology, Division of Infectious Agents and Cancer, University of Maryland School of Medicine, Baltimore, MD, United States; ^4^School of Specialization in Food Science, University of Rome “Tor Vergata”, Rome, Italy; ^5^Section of Clinical Nutrition and Nutrigenomics, Department of Biomedicine and Prevention, University of Rome “Tor Vergata”, Rome, Italy; ^6^National Institute for Cardiovascular Research (INRC), Bologna, Italy; ^7^Scientific Director, Istituto Nazionale Tumori Fondazione G. Pascale – IRCSS, Naples, Italy

**Keywords:** PI3K, COVID-19, inflammation, therapy, SARS-CoV-2

## Abstract

The spread of the novel human respiratory coronavirus (SARS-CoV-2) is a global public health emergency. There is no known successful treatment as of this time, and there is a need for medical options to mitigate this current epidemic. SARS-CoV-2 uses the angiotensin-converting enzyme 2 (ACE2) receptor and is primarily trophic for the lower and upper respiratory tract. A number of current studies on COVID-19 have demonstrated the substantial increase in pro-inflammatory factors in the lungs during infection. The virus is also documented in the central nervous system and, particularly in the brainstem, which plays a key role in respiratory and cardiovascular function. Currently, there are few antiviral approaches, and several alternative drugs are under investigation. Two of these are Idelalisib and Ebastine, already proposed as preventive strategies in airways and allergic diseases. The interesting and evolving potential of phosphoinositide 3-kinase δ (PI3Kδ) inhibitors, together with Ebastine, lies in their ability to suppress the release of pro-inflammatory cytokines, such as IL-1β, IL-8, IL-6, and TNF-α, by T cells. This may represent an optional therapeutic choice for COVID-19 to reduce inflammatory reactions and mortality, enabling patients to recover faster. This concise communication aims to provide new potential therapeutic targets capable of mitigating and alleviating SARS-CoV-2 pandemic infection.

## Introduction

At the end of 2019, a new infectious disease with some morbidity and mortality in humans emerged in Wuhan, China (1). The causative agent is a new coronavirus (CoV), later named SARS-CoV-2, and its related pathology has been classified as CoronaVirus Disease 2019 (COVID-19) (2, 3). On March 11, 2020, the World Health Organization (WHO) declared this infection a world pandemic (4). A total of 114 countries announced that 118,000 people had contracted COVID-19, and almost 4,300 had died (4, 5).

In general, COVID-19 does not represent a severe disease, but, in some people (usually the elderly and/or those with comorbidities), it can lead to pneumonia, acute respiratory distress syndrome (ARDS), and multi-organ dysfunction. Many data suggest that four fifths of cases are asymptomatic (6). The case fatality rate is estimated to be around 4.7% (data spread by the World Health Organization, WHO (5).

Coronaviruses are typically genomically shaped, positive-wrapped, single-stranded RNA viruses ranging from 26 to 32 Kb. There are currently four genera of known coronaviruses: alpha-CoV, beta-CoV, gamma-CoV, and delta-CoV (7).

Among mammals and birds, coronaviruses are responsible for many illnesses. In humans, often non-lethal diseases of the respiratory tract occur, as in the case of common cold, but sometimes coronaviruses generate highly lethal situations like pneumonia and bronchitis. Electively infecting the upper and lower airways and lungs, the SARS-CoV-2 virus causes breathing complications and acute respiratory failure syndrome (4, 8, 9). SARS-CoV-2 was isolated from human epithelial respiratory cells, sequenced and recognized as belonging to the beta-CoV group (10). The pathophysiology of SARS-CoV-2 is not yet well understood, but it is likely that it mimics that of SARS-CoV.

The angiotensin 2 converting enzyme (ACE2) has been identified as the candidate receptor for SARS-CoV-2 (3, 11), and an upregulation of ACE2 could improve SARS-CoV-2’s ability to infect target cells, such as the alveolar cells, increasing the risk of infection and related respiratory problems (8, 11). Acute lung damage is primarily the result of a major inflammatory cascade caused by virus replication (12).

Significant increase in pro-inflammatory cytokine levels, such as interleukin (IL)-12, IL-1, and IL-6, as well as chemokines, including the chemoattractant monocyte-1 protein (MCP-1), IL-8, and IFN-α-induced protein 10 (IP-10), have been reported (13). In addition, patients with severe and progressive disease reported higher plasma levels of IL-2, IL-6, granulocyte colony-stimulating factor (G-CSF), IP-10, IL-10, IL-7, MCP-1, and tumor necrosis factor (TNF)-α, suggesting a disruption correlated with the severity of the disease over the entire cytokine range (8).

Taking into consideration the importance of the immune balance in SARS-CoV-2 infection, multiple immune modulating drugs are being investigated for their effectiveness in COVID-19 therapy.

Some conventional synthetic disease-modifying anti-rheumatic drugs (scDMARDs) have been tested. Examples of these include Chloroquine, Glucocorticoids, Leflunomide, and Thalidomide, which interact with ACE2 to prevent invasion and replication of the virus, suppressing the immune and inflammatory responses (14, 15). Other biological disease-modifying anti-rheumatic drugs (bDMARDs), such as Tocilizumab and Anakinra, are under investigation since they are able to block the downstream signaling of IL-6 and IL-1 receptors, respectively (14, 15). In addition, Baricitinib and Ruxolitinib, targeted syntetic disease-modyfing anti-rheumatic drugs (tsDMARDs), have been tested as they selectively inhibit JAK1 and JAK2 kinase activity reducing the hyper-inflammation observed in COVID-19 (15, 16). Other immunotherapy approaches are under clinical review, such as stem cell therapy and convalescent plasma therapy (15), and ongoing trials are evaluating the effectiveness of these therapies (14, 15).

In this clinical scenario, patients with COVID-19 are characterized by increased T cells, natural killer (NK) cells, and monocyte-driven acute respiratory distress syndrome (ARDS). Many of these cells are regulated by phosphoinositide 3-kinase (PI3Kδ), which has recently been identified as contributing to multiple physiological and pathological processes of the respiratory tract, becoming a target for inflammatory and infectious lung diseases. The PI3Kδ isoform is particularly involved in the regulation of innate and adaptive immunity and inflammatory processes. A mutation that predisposes to a susceptibility to recurrent respiratory infections and bronchiectasis has been established by Angulo et al. (17) to illustrate the near association between PI3Kδ and lung infections.

Recent evidence has confirmed the use of PI3Kδ inhibitors in lung diseases (18, 19), and a preliminary clinical trial investigating the pharmacological and clinical properties of Nemiralisib (GSK2269557), performed by GlaxoSmithKline (GSK), is currently ongoing (20). In addition, a clinical trial on the use of Ebastine (antihistaminic of second generation that has been shown to be an effective treatment for both seasonal and chronic allergic rhinitis) in association with an antiviral in the treatment of COVID-19 positive patients is underway (21).

Given that serious and uncontrolled lung inflammation is likely to be a major cause of death in COVID-19, this review aims to evaluate an alternative therapeutic strategy by using the PI3Kδ inhibitor Idelalisib in association with Ebastine, which may successful inhibit the response of T cells and the release of pro-inflammatory cytokines (22).

## Epidemiological Data, Obesity and SARS-Cov-2

**FIGURE 1:**
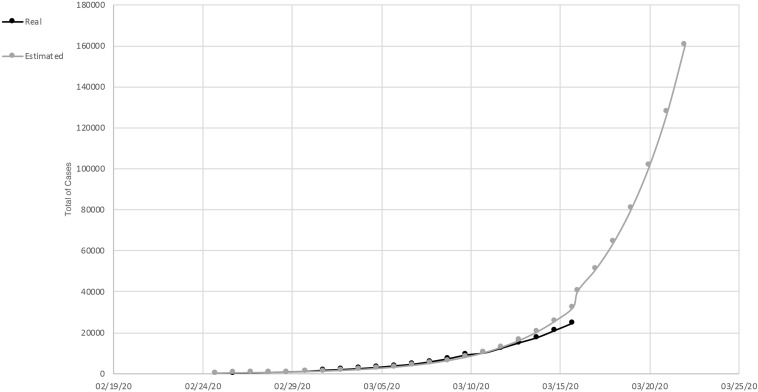
Total of cases of SARS-Cov-2, in Italy. Measured curve in black; predicted (exponential trend) in gray.

In March (March 15, 2020) an exponential increase in infections was detected in Italy. There was an increase of 2,853 cases compared to the previous day. Until that moment, 2,335 people were discharged and, unfortunately, 1,809 died; 20,603 people were found positive. Compared to the total number of cases reported on this date, 24,747, about 46% were hospitalized, and about 7% were in intensive care. Approximately 37% were isolated at home. The curve of the total number of cases/real curve of total cases slowed down growth over the last 3 days compared to the estimated exponential trend (Figure 1). This is likely due to the very first effect of the containment measures implemented on March 9. In fact, the cases doubled every 4 days.

In Lazio Italian Region, an example of a region in which people did not adopted the containment measures in an optimal way, the total number of cases recorded was 436. Of these, 58% were in hospital, 31% were in intensive care, and 16% of the total cases died. The estimated and actual curves of the total number of cases was still superimposable. In the number of cases, a very slight deflection was observed. They doubled not every 2 days but every 3 (Department of Civil Protection, COVID-19 Italy).

On July 7, cumulative data regarding the epidemic curve of COVID-19 in Italy showed a total of 241,550 cases and a case-fatality ratio of 14%; the gender percentage was 45.6% for males and 54.2% for females, and the median age of affected patients was 61 years (data spread by the COVID-19 Task force of the Department of Infectious Diseases and the IT Service *Istituto Superiore di Sanità*, Italy). The global situation counted 11,425,209 confirmed cases with a case-fatality ratio of 4.7% (data spread by the World Health Organization, WHO). Evidence indicates that, worldwide, both sexes may be equally affected, even if the number of deaths in men is higher than in women, indicating clear gender differences, likely related to the influence of sex hormones on ACE2 expression (23).

Characteristics of COVID-19 subjects have shown that the virus mainly affects older age groups and that pre-existing pathologies, in particular obesity, are the primary cause of admission (24, 25); Gualtieri et al. observed that 57% of patients are obese (26). Despite widespread data on comorbidities, there has been an increase in the consumption of ultra-processed products, particularly dairy products, during quarantine (27). Future and current scientific literature will have to stem the quality of nutrients from poor nutrition and obesity (27).

## COVID-19 and Neurological Signs

SARS-CoV-2 entry into human host cells is predominantly regulated by the cellular receptor ACE2, expressed in human airway epithelia, lung parenchyma, vascular endothelia, kidney cells, and small intestine cells (28). Nevertheless, the presence of ACE2 is not sufficient to make host cells vulnerable to infection; in addition, some ACE2-expressing endothelial cells and lines of the human intestinal cells were not infected with SARS-CoV-2 (29), whereas some cells, such as hepatocytes, may be infected without detectable levels of ACE2 expression. Furthermore, the infection was also reported in the central nervous system (CNS), particularly in the brainstem (BS), which plays a key role in the control of the respiratory and cardiovascular system. Coronavirus infection of human CNS seems to be restricted to neurons and the lack of inflammation in infected brains raises the possibility that neurons die by apoptosis in mice (30). Specific sample studies from SARS patients have shown the presence of SARS-CoV particles in the brain, mainly in the neurons (31). Evidence, on the other hand, indicates that CoVs can first invade peripheral nerve terminals and then gain access to the CNS via a synapse-connected pathway. This neuroinvasive propensity has proven to be a common characteristic of CoVs. COVID-19 studies highlight the significant increase in pro-inflammatory factors (32) that can also be found in the brain; in particular, in animal models, caspase-1 activation causes higher cleaved caspase-3 levels with consequent activation of IL-1β. There is a possibility that the activation of the NF-kB pathway may lead to a transcriptional up-regulation of the NLRP3 inflammasome, pro-IL-1β, or pro-IL-18 inactive forms (33). By forming a complex pro-caspase-1 oligomer, NLRP3 accounts for its conversion into the active form of caspase-1 and, subsequently, the cleavage of pro-IL-1β plus pro-IL-6 into its active forms, thereby triggering inflammatory brain conditions (34). These indications suggest the possible involvement of microglia, and, in particular, the shift from M2-like polarization to M1-like polarization may be responsible for the prompt expression of pro-inflammatory mediators and, subsequently, the recruitment of circulating monocytes (35). In the specific scenario of COVID-19, there is an increase in infiltration of innate immune cells and an increase in cerebral pro-inflammatory factors. These factors appear to maintain the infection of COVID-19 by activating macrophages, changing the brain’s homeostasis.

## Intriguing PI3Kδ Involvement and Controversial Role of ACE Inhibitors and Angiotensin II Type-I Receptor Blockers

PI3Kδ is an enzyme able to catalyze the phosphorylation of phosphatidylinositol 4, 5-bisphosphate generating phosphatidylinositol 3,4,5-triphosphate, a crucial player of immune cell activation, proliferation, and differentiation [Down et al. (36) and references therein]. Being predominantly expressed in leukocytes, PI3Kδ is considered a promising therapeutic target in inflammatory diseases, such as severe respiratory conditions and rheumatoid arthritis (36, 37). In fact, both effector and memory T cells use p110δ, the catalytic domain of PI3Kδ, to promote the production and the release of inflammatory cytokines, such as interleukins and interferon-γ (38).

Interestingly, SARS-CoV-2 is supposed to mainly act through T lymphocytes, inducing high levels of inflammatory cytokines and producing, in turn, fatal inflammatory responses and acute lung injury (39). In this context, targeting PI3Kδ could be of high interest to slow down the dysregulation of the immune response visible in COVID-19 patients. Administration of the drug via inhalation may be required in order to prevent a harmful effect based on a systemic inhibition of PI3Kδ. Since already tested to treat respiratory diseases, selective PI3Kδ inhibitors may be considered as a therapeutic drug also in COVID-19 (36, 40). The possibility of decreasing IL-6, an independent prognostic marker of cardiovascular diseases (CVDs) (41), by blocking PI3Kδ could represent an additional protective strategy since SARS-CoV-2 also causes acute myocardial injury and chronic damage to the cardio-vascular system (42). Indeed, CVDs represent a significant co-morbidity in patients diagnosed with COVID-19 together with diabetes and hypertension, indicating that subjects with cardiovascular problems are at higher risk (43–45). The comorbidities in these patients are often associated with ACE inhibitor (ACEI) treatments (46), which, together with angiotensin II type-I receptor blockers (ARBs), represent the cornerstone of the clinical management of patients with hypertension and diabetes, crucial cardiovascular risk factors (47). Thus, a possible connection between the use of ACEIs and COVID-19 can be supposed, especially considering the role of ACE2 that acts as a functional SARS-CoV receptor (48). Moreover, recently it has been shown that SARS-CoV-2 also uses ACE2 as a cellular receptor to enter the target cells [Zhou et al. (3), Moccia et al. (49) and references therein]. Preclinical studies have shown that selective ACEIs and ARBs, either alone or in combination therapy, can induce significant upregulation of cardiac ACE2 mRNA and an increase in plasma soluble ACE2 (50). The current hypothesis is that the ACEI-induced upregulation of ACE2 could improve SARS-CoV-2’s ability to infect target cells, raising the risk of SARS-CoV-2 infection. However, there is great concern about this issue: some authors (46, 51) recommend alternative therapies in patients with cardiac disease, hypertension, or diabetes exposed to ACE2-increasing drugs, whereas other scientists recommend that such therapies should not be suspended due to the COVID-19 infection in the absence of epidemiological evidence. In this regard, the Council statement of the European Society of Cardiology (ESC) noted “the lack of evidence supporting the harmful effects of ACEI and ARB in the context of the outbreak of the COVID-19 pandemic.” Therefore, the problem related to replacing ACEIs and ARBs, in the case of hypertension, remains controversial in patients with COVID-19. Further studies are required to confirm the link between these observations and to investigate the clinical outcome in COVID-19 patients with cardiovascular complications under ACEIs and ARBs.

An additional therapeutic target related to the PI3K molecular pathway may be represented by the Janus kinase (JAK), an immediate upstream actor of this cascade known to regulate inflammatory cytokine [Seif et al. (52) and references therein]. Several authors suggest the use of JAK inhibitors (JAKi) in the treatment of COVID-19 as in the case of Baricitinib, a JAK inhibitor targeting JAK1 and JAK2, mechanistically able to inhibit the signaling of many COVID-19-related cytokines (52). However, in patients with CVDs, the treatment with JAKi for long periods is not recommended, as their use increases the thromboembolic risks already present in COVID-19 patients (53). At the moment, clinical evidence does not provide any assurance about JAKi treatment, and additional studies are thus needed.

## Therapeutic Potential of PI3Kδ Inhibitors and Ebastine in COVID-19

Increasing data point on PI3K pathways as anti-inflammatory targets in airway diseases, including asthma, allergic rhinitis, chronic obstructive pulmonary disease (COPD) and others. PI3Kδ, whose expression is restricted to leukocytes, is thought to be a valuable treatment in inflammatory lung disorders (54). PI3Kδ knockout and kinase-dead knocking (KI) mice display reduced T-cell response, similar to IC87114-treated mice, and a selective PI3Kδ inhibitor, as visible from the reduction in the clonal expansion of CD4^+^ T cells in the presence of antigens (55).

PI3Kδ inactivation also affects the role of mast cells with respect to proliferation, under stem cell factor (SCF) or IL-3 stimulation, to degranulation, and regarding the downstream cytokine release of FceR1 (56). Mast cells are the main effectors in experimental models of both asthma and allergy, and they are mitigated by the inhibition of PI3Kδ using IC87114 (57). In addition, PI3Kδ is also supposed to play a role in the adhesion of neutrophils to the vascular endothelium induced by TNF-α, which makes its inhibition a possible therapy for lung diseases (58). PI3Kδ inhibition may prevent the release of inflammatory molecules reducing the recruitment of T lymphocytes and neutrophils. The inhibition of the PI3Kδ cascade may also improve macrophage- and neutrophil-mediated bacterial clearance, resulting in a decreased incidence of pathogen-induced exacerbations.

PI3Kδ signaling is crucial for the survival, migration, and activation of B cells as well as for their downstream antigen receptor (BCR), co-receptor CD19, and activation/costimulatory receptors, i.e., CD40 and Toll-like receptors (TLRs). B cells are the first line of humoral immunity and the formation of antibodies in response to COVID-19 (59). It is assumed that in some patients treated for granulomatosis with polyangiitis or for chronic rheumatic diseases, the therapy does not worsen the clinical situation and attenuates its severity (60, 61). However, within the context of the aberrant production of inflammatory cytokines triggered by SARS-CoV-2, drugs acting on B cells and antibody production may have detrimental result (60, 61).

Clinical and *in vivo* studies for the treatment of B-cell malignancies and chronic graft-versus-host disease (cGVHD) have shown that the use of Ibrutinib can abrogate pulmonary inflammatory cytokines and lung injury since patients diagnosed with Waldenstrom macroglobulinemia who are treated with Ibrutinib and are positive for COVID-19 seem to be protected against lung diseases (62–64). More recently, a clinical study supported the anti-inflammatory ability of PI3Kδ inhibitors in a cohort of COPD patients, showing reduced IL-8 and IL-6 levels into the sputum (65).

Idelalisib is currently marketed for the treatment of certain patients with follicular lymphoma, small lymphoma, and chronic lymphocytic leukemia B-cell malignancies. In the current study, the tolerance to the toxic effects of Idelalisib may be improved by shortening the treatment period respect to that used in oncologic patients, that usually received Idelalisib for considerably longer stages. A small evidence of the biology study with Idelalisib was conducted in patients with allergic rhinitis, an inflammatory-driven upper airway disease predominantly associated with type 2T helper cell (TH2) (66). These investigators demonstrated that inhibition of PI3Kδ by Idelalisib significantly reduced total scores of nasal symptoms, nasal airflow, weights of nasal secretion and congestion. Idelalisib effectively reduced allergic rhinitis symptoms in a 4-h environmental allergen challenge in the Vienna Challenge Chamber (VCC), and it performed higher than placebo regarding the primary efficacy endpoint of change from baseline Total Nasal Symptom Score (TNSS) and for some secondary efficacy endpoints, including Total Symptom Scores (TSSs) and average nasal airflow. Allergy medicines (e.g., corticosteroids, antihistamines, and anticholinergic medicines) were banned during the study. An *ex vivo* full-blood basophil (CD63) activation assay confirmed that PI3Kδ was engaged in their study performing observation/measurement periods. McLeod and collaborators identified the potency and selectivity of four *in vitro* enzyme and cellular assay inhibitors (MSD-496486311, MSD-126796721, Idelalisib, and Duvelisib) (67). Cytokine imbalance and cell migration to inflammatory tissues are features of the lung damage observed in severe COVID-19; the same condition is also evident in allergic diseases, where the activation of pro-inflammatory cells plays a key role in the inflammatory response.

The possible pharmacological action of Ebastine in COVID-19 is based on its ability to inhibit T-cell activation, migration, and pro-inflammatory cytokines release, as demonstrated in allergic diseases (68). The common point between COVID-19 and allergic diseases makes Ebastine a potential therapeutic choice and inhibiting the release of both TNF-α and granulocyte-macrophage colony-stimulating factor (GM-CSF). The macrophages represent the predominant cellular portion in the airways and one of the most abundant cells in the lung parenchyma; they can synthesize and release a wide spectrum of pro-inflammatory cytokines, and their inhibition is an effective strategy for mild or serious lung disease. Our hypothesis is supported by a recent clinical trial conducted at Mianyang Central Hospital (n°ChiCTR2000030535), sponsored by the Wuhan Red Cross Hospital Ethics Committee (21).

A detailed analysis of epidemiological evidence linked to extreme acute respiratory syndrome COVID-19 showed a rise in autoimmune, auto-inflammatory diseases, including pediatric inflammatory multisystem syndrome (PIMS) and children’s inflammatory multisystem syndrome (MISC) (69). Clinical evidence has highlighted how COVID-19 affects children in a milder manner than adults but with different symptoms, closer to PIMS or Kawasaki-like disorder (KD). These subjects display high levels of inflammatory biomarkers, such as reactive protein C, pro-calcitonin, and ferritin, together with large numbers of leukocytes and neutrophils as well as with the classical intestinal and respiratory symptoms of KD; they also have high frequency of myocarditis and pericarditis together with thrombocytopenia (70). These patients show elevated concentrations of IL-6 as a common trait, which is similar to what is seen in adults with COVID-19 who are severely ill. In this scenario, a treatment based on Idelalisib and Ebastine might be conceivable, suppressing the inflammatory cascade, as also suggested by Galeotti et al. (71) for treatment with Sarilumab or Tocilizumab.

Histamine, primarily via the H1-receptor, is an important mediator of allergy symptoms. H1-antihistamines, which stabilize the receptor in its inactive form, are the preferred treatment for some chronic allergic conditions. Ebastine is a well-established oral second generation H1-antihistamine administered once daily. Recent evidence (72) identifies, through a computational docking analysis, a possible anti-COVID molecule in the chemical structure of Ebastine, supporting in-depth clinical studies for its use.

Currently, Idelalisib is used to treat patients with B-cell lymphoma, particularly relapsed indolent lymphoma, as described by Cassaday et al. (73). The related adverse reactions reported for Idelalisib are fatigue (30%), nausea (30%), diarrhea (43%), pneumonia (11%), neutropenia (56%), elevated transaminases (47%). Idelalisib is reported to have major adverse reactions when associated with other chemotherapy drugs (72).

Considering the inflammatory pathways involved in the COVID-19 pulmonary disease, we suggest a possible therapeutic role for Idelalisib (PI3Kδ inhibitor) alone or in combination with Ebastine.

## Synopsis and Conclusion

Since January 30, 2020, when the WHO declared the epidemic of COVID-19 as a public health emergency of international concern, the attention of the world, especially in terms of clinical research, has been strongly addressed to the better understanding of the novel SARS-CoV-2 and its related disease (74).

The novel coronavirus uses ACE2 as a receptor to enter the host cells and mainly affects the respiratory tract. SARS-CoV-2 shows a high human-to-human transmission, and its clinical symptoms include fever, cough, fatigue, and, rarely, gastrointestinal infection. Old age and the presence of comorbidities make people more susceptible to the infection and to its adverse prognosis, with a higher risk of death (74).

COVID-19 generates a general increase in inflammatory cytokines, such as IL-1β and IL-6, and a mortality rate of 7.5% (75). SARS-CoV-2 may affect both the upper and lower respiratory tract, causing a mild or highly acute respiratory syndrome. The binding of SARS-CoV-2 to the Toll Like Receptor (TLR), results in the release of pro-IL-1β, which is cleaved by caspase-1, producing active mature IL-1β that in turn mediates lung inflammation.

The inhibition of IL-1 family members and of IL-6 is used as a therapeutic strategy in many inflammatory diseases, including viral infections (76). In Italy, Ascierto et al. tested a specific drug, namely Tocilizumab, in COVID-19 patients obtaining very encouraging results. Tocilizumab, a monoclonal antibody capable of inhibiting IL-6, has resulted in a clear improvement of health in patients affected by COVID-19, although not all patients were sensitive to this treatment. On the basis of this scientific evidence, our hypothesis is that patients who are not sensitive to Tocilizumab may have a progressive and more aggressive systemic inflammation that requires another drug capable of acting selectively on inflammatory cascades (72).

Even if anti-viral therapy still remains a treatment option, when COVID-19 presents with a cytokine storm as a major immunological complication, antivirals are no longer sufficient and have to be combined with proper anti-inflammatory drugs (77). In the most severe stage of the disease (stage 3, as suggested by Siddiqu and Mehra) (78) especially, systemic inflammation, in several cases, can be overcome by the use of immunomodulatory drugs (79). Currently, many anti-inflammatory therapies have been used to counteract COVID-19 cytokine storm. Glucocorticoids, chloroquine/hydroxychloroquine, immunosuppressants, non-steroidal anti-inflammatory drugs, cytokine antagonists (i.e., JAK and TNF inhibitors, IL-6R monoclonal antibodies, and IL-1 antagonists), and intravenous immunoglobulin (IVIG) administration are emerging as promising tools (79).

**FIGURE 2:**
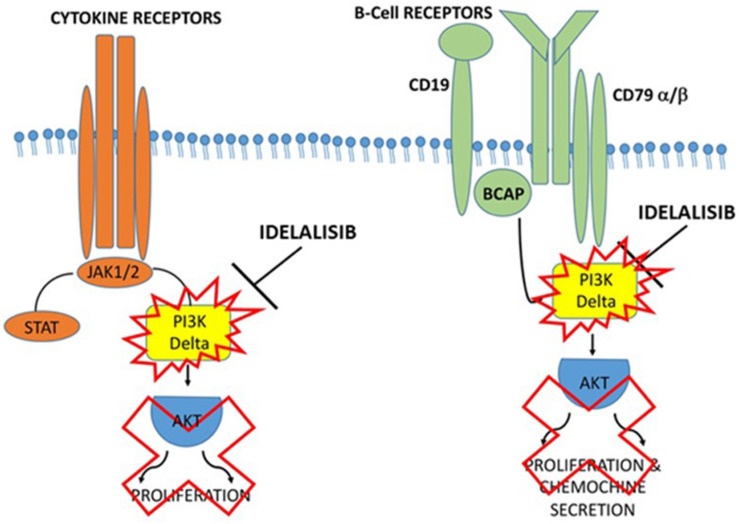
Proposed action mechanism of Idelalisib in PI3Kδ inflammatory cascade. Supposed mechanism used by Idelalisib to stop the inflammatory machine activation by blocking PI3Kδ in COVID-19 infection.

Idelalisib may have a potential key role in suppressing the entire inflammatory cascade and preventing the release of more inflammatory mediators. Inhibiting the PI3Kδ pathway could reduce the incidence of macrophage infiltration and improve the patient’s condition. Inhalation of Idelalisib may result in suppression of Caspase-1, IL-1, and IL-6 by blocking PI3Kδ and may therefore have a strong anti-inflammatory effect on COVID-19 (Figure 2).

The treatment of ACE2 inhibitors may be another possible therapeutic approach, but the data are controversial and further investigations are needed. For this reason, at this time, the ACE2 inhibitor approach does not appear to be the best therapy for COVID-19. Together with PI3Kδ inhibitor Idelalisib, Ebastine treatment in COVID-19 also appears to be an excellent inhibitory drug approach to T-cell pro-inflammatory cytokines IL-6 and TNF-α as demonstrated in allergic diseases (67, 68). Ebastine may cause improvement in the patient’s breathing with COVID-19 pulmonary symptoms.

The balance between the benefits and limitations of these treatments is still under discussion, in particular with regard to the choice of eligible patients, the appropriate drugs, the onset and duration of treatment. As reviewed by Zhang et al. (79), anti-inflammatory drugs may increase the risk of secondary infections, may act too specifically on few inflammatory targets, and may sometimes block other critical modulatory pathways. Several studies have shown that PI3K pathways are key anti-inflammatory target for many lung diseases (79). However, in the context of COVID-19, it is possible to speculate that inhibiting PI3Kδ may reflect the same dilemma that still exists for other anti-inflammatory drugs and that, for this reason, its therapeutic eligibility should be discussed, taking into account the pathological characteristics of each individual patient; that its route of administration should be chosen in order to avoid systemic toxicity (40).

In conclusion, taking into account the scientific evidences on the inflammatory pathways involved in COVID-19, we suggest a potential therapeutic approach with Idelalisb alone or in combination with Ebastine in patients suffering from COVID-19, in order to alleviate the harmful and sometimes fatal pulmonary disease associated with this pandemic viral infection.

## Author Contributions

GP and TP arranged and designed the manuscript. GS and EA developed and performed the tests and wrote the manuscript. GS edited the manuscript. CR, AB, ADB, and PG arranged and designed the scientific approaches. ADL, TA, and GB contributed with the editing of the manuscript. All authors participated with the data analysis and read and approved the final manuscript.

## Conflict of Interest

The authors declare that the research was conducted in the absence of any commercial or financial relationships that could be construed as a potential conflict of interest.

## Abbreviations

ACE2: angiotensin-converting enzyme 2
ACEIs: ACE inhibitors
ARBs: angiotensin II type-I receptor blockers
BS: brainstem
CNS: central nervous system
CoV: novel coronavirus
COVID-19: Coronavirus Disease 2019
CVDs: cardiovascular diseases
IP-10: induced protein-10
KI: kinase-dead knocking
MCP-1: monocyte chemoattractant protein-1
PI3K δ: phosphoinositide 3-kinase δ
SARS-CoV-2: human respiratory coronavirus
SCF: stem cell factor
TH2: T helper 2 cell
TLR: Toll like receptor
WHO: World Health Organization.
